# Daily Eating Window and Obesity Markers in a Sample of Schoolchildren from Vienna: Insights from the EDDY Study

**DOI:** 10.3390/nu17101661

**Published:** 2025-05-13

**Authors:** Paula Moliterno, Victoria Donhauser, Kurt Widhalm

**Affiliations:** 1 Austrian Academic Institute for Clinical Nutrition, 1090 Vienna, Austria; paula.moliterno@eddykids.at (P.M.);; 2 Division of Clinical Nutrition and Prevention, Department of Pediatrics, Medical University of Vienna, 1090 Vienna, Austria

**Keywords:** eating window, meal timing, childhood obesity, Vienna

## Abstract

Background/Objectives: The eating window concept has been understudied in children, with no reports from Austria. This study explored meal timing and its association with obesity-related variables in a sample of Viennese schoolchildren. The effect of a healthy intervention on obesity variables according to the daily eating window was assessed. Methods: The EDDY study included 138 third-grade students from three Vienna schools. Baseline meal timing was assessed using self-administered questionnaires, and the daily eating window—the time between the first and last meal—was calculated and categorized into tertiles. Anthropometric and body fat measurements were taken at baseline and after 21 months. Baseline outcomes were compared between children with long (LEW; 3rd tertile) and short (SEW; 1st tertile) eating windows using adjusted linear regression analysis. Longitudinal changes were analyzed using mixed models for repeated measures. Results: The median age was 7.9 years, and 26.8% were classified with overweight/obesity. The children’s eating window spanned 11:40 h, from 7:00 to 19:00. More than half (52.2%) reported fasting 1–2 h before bed. Children had four daily meals; 16.4% skipped breakfast, while 51.5% ate it regularly. Meal timing variables did not differ by weight status. Children with a LEW (≥12:05 h) had lower BMI-SDS (−0.66) and fat mass index (−1.06) than those with a SEW (≤11:05 h). No longitudinal changes in BMI-SDS, fat mass index, or waist-to-height ratio were observed across eating window tertiles following the intervention. Conclusions: In a non-representative sample of Viennese children, the eating window ranged from 9:30 to 13:30 h, similar to Austrian adults but differing from other Western European countries.

## 1. Introduction

Achieving a healthy lifestyle and body weight maintenance is a challenging task that can be acknowledged by the extensive rates of overweight and obesity, even in the pediatric population. Projections for Austria indicate a 1.6% annual growth rate in the number of children with high body mass index (BMI) between 2020 and 2035 [1]. Nutritional status results from a complex interaction between the energy intake and macronutrient composition of a person’s food, the practice of physical activity, and each genetic input.

However, this oversimplified equation has proven to be influenced by several other factors, such as physiological, cultural, or individual characteristics that, until recent years, were not studied in depth. The conjunction of those factors can contribute to obesity, as they can induce alteration of the biological mechanisms that maintain normal mass and function of the adipose tissue. As highlighted recently by a Commission that established a new obesity model [2], the causes of obesity remain incompletely understood. In this sense, the circadian clock, an endogenous timing system that drives biological functions and behavioral outputs in approximately 24 h, has been outlined as central to metabolic processes [3]. It comprises an internal clock, which is mainly regulated by light, and several peripheral clocks, which are highly influenced by the central clock and external factors, such as (but not limited to) food intake and its characteristics. Meal timing, as one dimension of feeding behavior, is considered to be one key factor influencing the circadian clock, as it can entrain the peripheral clocks of tissues and organs related to food intake, such as the liver and pancreatic tissue [4]. The mechanisms underlying the connection between meal timing and cardiometabolic health remained unclear as the effect cannot be solely attributed to mistiming in meals but also to accompanying changes, such as caloric intake, duration of fasting, or frequency of meals [4]. In adults, epidemiological data showed that an eating duration longer than 12 h was associated with a higher prevalence of abdominal obesity compared to those who ate their meals in a shorter eating duration (≤12 h) [5]. However, the importance of chrono-nutrition variables in children’s health has not yet been well studied [6,7]. A recent systematic review found a usual daily average eating window in children and adolescents of 11.3 h. However, the authors acknowledge that reported data on daily eating windows among studies was variable. Therefore, more research on this topic is suggested [7]. Moreover, no description of meal timing variables has yet been reported in Austrian children, which could vary from the meal timing patterns recently described for adults [8].

Our study aimed to describe the daily eating window in a non-representative sample of school children from Vienna and to assess its association with meal timing and obesity-related variables. Additionally, we aimed to evaluate the impact of a healthy nutritional and physical activity intervention on BMI-SDS, total body fat, and abdominal body fat according to the daily eating window.

## 2. Materials and Methods

### 2.1. Study Sample

The sample belongs to the EDDY (“Effect of sports and diet training to prevent obesity and secondary diseases and to influence young children’s lifestyle”) program, which started in September 2022, with support from the Federal Ministry of Education, Science and Research of Austria, proposing a healthy nutrition and physical activity intervention in one middle school (school 1) from Vienna, Austria. Two other schools selected by the Federal Ministry acted as controls: one included an external government-funded physical activity proposal but no nutritional education (school 2), and the other received no intervention at all and just followed the regular curricula (school 3). The three middle schools included were in Vienna’s 23rd, 10th, and 12th districts. Regarding the districts’ socioeconomic status, the 23rd district boasts an average net income slightly above the city and national averages [9], contrary to the 10th and 12th districts, which rank among those with lower and mid-range average incomes. The 23rd district has a comparatively low percentage of residents with academic degrees, although it stands out with one of the highest percentages of residents holding vocational training qualifications. The 10th and the 12th both exhibit lower rates of individuals with academic degrees than other Viennese districts [9].

The intervention included a multi-component approach of two additional sports units and nutritional education lessons of 90 min each per month. The nutritional education comprised diverse topics related to healthy eating and well-being; however, it did not include content related to eating in a specific time frame during the day. At the end of the second school year (June 2024), the last measurements after the intervention were taken. All children with anthropometric data at the beginning and end of the study, and parent questionnaire information at baseline, were selected. From n = 223 children selected to participate in the longitudinal study, n = 138 (61.9%) children with complete data on the outcome variables were included. Reasons for exclusion included absence of parental consent to participate or missing measurements at baseline or the end of the program. Finally, school 1 included n = 48 [8.1 (7.2–9.1) years] children, school 2 n = 52 [7.8 (7.2–9.0) years], and school 3 n = 38 [8.1 (7.3–9.6) years]. The general characteristics of the participants according to the school group are shown in Table S1. The overall sample at baseline was used to describe the daily eating window and meal timing variables cross-sectionally at baseline. To study longitudinal changes in anthropometric and body composition variables according to the baseline tertiles of the daily eating window after the healthy nutrition and physical activity intervention, we assessed changes in the school that received a nutrition and physical activity intervention (school 1), compared to the school that only received a physical activity intervention (school 2), or no intervention at all (school 3).

Ethical approval was obtained from the Ethics Committee of Sigmund Freud University, Vienna (PAFGRW9O@EFQV885378—15 November 2016). Parents provided written consent for their child’s participation in this study. Older children also gave written assent for their participation. Anonymization was assured during the data collection process.

### 2.2. Meal Timing Variables

Meal timing data used in this study included the variables eating window, first and last meal hours, number of meals per day, pre-bed fasting time, and breakfast frequency, and were assessed through questionnaires at baseline. Meal information for each eating episode and time was self-reported. The eating window was calculated based on the hours between each child’s first and last meal, and subsequently, the participants were divided into tertiles according to the variable distribution for the overall group to classify eating window status into “short” (SEW), “moderate” (MEW), or “long” (LEW). The number of meals corresponded to an average reported daily intake of meals and snacks. To report pre-bed fasting time, parents were asked to select from the categories “≤1 h”, “1–2 h”, “2–3 h”, and “≥3 h” according to how long (on average) the amount of time between the last meal and the bedtime fast was. The frequency of having breakfast was assessed during weekdays and weekends and further categorized as “regular” breakfast intake when a participant declared they always had breakfast at home on all weekdays and weekends. If breakfast was skipped some days (or permanently) during the week and weekend, we categorized a participant as having “irregular” breakfast intake.

### 2.3. Anthropometric and Body Composition Variables

Trained personnel measured weight, height, waist circumference, and body fat under standardized procedures at baseline and after 21 months. Children’s body weight was measured using a Tanita body composition electronic scale (MC-780MA, TANITA Corporation, Tokyo, Japan), and height was measured using a stadiometer (SECA 213, Hamburg, Germany), with the child standing without shoes. Body mass index (BMI) (kg/m^2^) was calculated and transformed to age- and sex-specific percentiles to classify nutritional status [10]. Low weight was classified as <3rd percentile, normal weight ≥3rd percentile, and overweight ≥90th percentile. The obesity category was considered as the sum of the original categories of obesity (≥97th percentile) and extreme obesity (≥99.5th percentile) [10]. As only one child was classified as low weight, we combined the low with the normal weight category. A binary categorization of weight status (normal weight; overweight/obesity) was used to assess the relationship between meal timing variables and weight status. The BMI-SDS was calculated using the LMS method [10]. Following standardized procedures, total body fat was obtained through body composition analysis (MC-780MA, TANITA Corporation, Tokyo, Japan). Total children’s body fat was assessed using the fat mass index, standardizing total fat mass (kg) to the square of height (m^2^). Waist circumference was measured using an inelastic tape (SECA 201, Hamburg, Germany) at the narrowest point between the last rib and the highest point of the iliac crest. The anthropometric index waist-to-height ratio (waist—cm/height—cm) was used to assess abdominal fat distribution.

### 2.4. Additional Variables

Socioeconomic variables such as age and sex were assessed through the self-administered questionnaires. Additionally, parents were asked to report their nationality, weight (kg), and height (cm). Furthermore, parental BMI (kg/m^2^) was calculated. As sleep is closely interrelated with the circadian system [11], we described the average quantity of sleep in hours per day, as assessed by parental reports through the questionnaires. Parents reported the amount of daily nocturnal sleep duration (and naps during the day) during school days (weekdays) and weekends, after which the average sleep duration was calculated as [(average sleep duration on weekdays × 5) + (average sleep duration on weekends × 2)] divided by 7.

### 2.5. Statistical Analysis

Data distribution was checked for normality using the Shapiro–Wilk tests. Since the data were found to be not normally distributed, nonparametric analyses were conducted. Descriptive analyses were performed by calculating medians, 95% confidence intervals (CI), and the frequency and percentage distribution. Descriptive analyses were used to summarize the participants’ general characteristics according to sex, school group, weight status, and the tertiles of the eating window. The differences across sex and weight status (normal weight vs overweight/obesity) were assessed using Chi-square (or Fisher) tests for categorical variables and the Mann–Whitney U nonparametric test for continuous variables. The differences across the school groups and the tertiles of the eating window were assessed using Chi-square (or Fisher) tests for categorical variables and the Kruskal–Wallis nonparametric test for continuous variables. When assessing differences between weight status categories and characteristics across the tertiles of the eating window, models were adjusted for age, sex, quantity of sleep, and school group, as demographic and behavioral outcome-relevant variables. To account for those potential confounding factors, we employed a rank-based analysis of covariance (ANCOVA) before using the Mann–Whitney U and Kruskal–Wallis test. The dependent variable and covariates were first transformed into ranks, which were then used to conduct nonparametric analyses across weight status or the tertiles of the eating window categories while adjusting for the covariables. The test of interaction between weight status and group and the tertiles of the eating window and group was nonsignificant. The statistical threshold for the interactions was set at *p* < 0.05. Post hoc pairwise comparisons between the tertiles of the eating window categories were conducted using Tukey’s test to adjust for multiple comparisons. To study the association between the tertiles of the eating window with BMI-SDS, fat mass index, and waist-to-height ratio, multivariable linear regression analysis was used to estimate the exposure coefficients and the CIs, adjusting for school group, sex, age, and sleep amount, as demographic and behavioral outcome-relevant variables.

Longitudinal changes in BMI-SDS, total body fat (assessed through the fat mass index), and abdominal body fat (assessed through the waist-to-height ratio) in response to a healthy lifestyle intervention were assessed using mixed models for repeated measures. The school group, time, tertiles of the eating window, and their interaction, were treated as fixed effects, and the participants were treated as random effects. The models were adjusted for age, sex, and the quantity of sleep as demographic and behavioral outcome-relevant variables. Data analysis was conducted using SAS (https://www.sas.com/en_us/software/on-demand-for-academics.html, accessed on 1 March 2025) OnDemand for Academics (Cary, NC, USA), and a *p*-value < 0.050 was assigned as significant.

## 3. Results

The median age of the total sample (n = 138) was 7.9 (95% CI: 7.2–9.3) years old, with no difference between girls and boys. The median BMI and BMI-SDS were 16.5 (95% CI: 14.0–25.2) kg/m^2^ and 0.29 (95% CI: −1.25–2.71), respectively. Thirty-seven children (26.8%) were classified with overweight/obesity. The waist-to-height ratio was 0.45 (95% CI: 0.40–0.56), and the fat mass index was 3.76 (95% CI: 2.48–9.53), being higher in girls than boys (*p* = 0.003). Regarding the meal timing variables, overall, the parents reported that their child’s daily eating (eating window) spanned 11:40 hours (95% CI: 9:30–13:30), with the first meal at 7:00 (95% CI: 6:10–9:30) and the last meal at 19:00 (95% CI: 18:00–20:30). More than half of parents (52.2%) reported that their child had a pre-bed fasting time of 1 to 2 h, while 19.6% had 1 h or less, and 5.1% had 3 h or more. The number of meals per day was four (95% CI: 3–6), which showed no difference between sexes (*p* = 0.947). Regarding breakfast, 22 (16.4%) parents reported that their children skipped breakfast during the week. During the weekend, only one participant reported skipping it, while the majority (98.5%) had breakfast on both days of the weekend. Overall, 51.5% of the children ate breakfast regularly, with no differences according to sex (*p* = 0.424). Total daily sleep duration was 9:47 hours (95% CI: 8:42–11:00). Fathers reported a median BMI of 27.7 (95% CI: 21.3–36.3) kg/m^2^, while that of mothers was 24.8 (95% CI: 20.0–36.5) kg/m^2^, being higher in mothers of girls compared to that of mothers of boys (*p* = 0.047). Overall, 63.2% of both parents reported being born outside Austria. The characteristics of the participants according to sex and school group are shown in Supplementary Materials (Tables S1 and S2, respectively). Overall, differences were observed in the percentage of children with obesity between school groups, being 8.3% in school 1, 9.6% in school 2, and 29.0% in school 3 (*p* = 0.050). The waist-to-height ratio (*p* = 0.017) and fat mass index showed differences, with the latter being lower in school 1 [3.37 (2.19–9.00)] compared to school 2 [3.66 (2.64–8.66)] and school 3 [4.14 (2.63–10.80)] (*p* = 0.009). Regarding the meal timing variables, differences were observed in the overall distribution of meals per day across school groups.

When the meal timing characteristics were studied according to weight status, no statistical differences were observed between children with normal weight and those with overweight/obesity, except for the overall distribution of meals per day (Table S3). A nonsignificant trend was observed in children with normal weight, with lower pre-bed fasting times than those with overweight/obesity (*p* = 0.081). Moreover, children with normal weight reported longer daily sleep duration than those with excessive weight (10:00 vs 9:30 h; *p* = 0.005).

Table 1 shows the characteristics of the participants according to the baseline tertiles of the eating window, representing those with short (1st tertile, ≤11:05 h—SEW), moderate (2nd tertile, 11:05–12:05 h—MEW), and long (3rd tertile, ≥12:05 h—LEW) eating window status.

The median eating window time was 10:30, 11:40, and 12:45 h for the short, moderate, and long eating window statuses, respectively. No baseline association was observed between eating window status and school group. Children reporting a LEW (≥12:05 h) had a lower BMI-SDS and fat mass index than those with a SEW (≤11:05 h). Regarding meal timing variables, those with a LEW had an earlier first meal at 7:00 (95% CI: 6:00–7:30) and a later dinner at 19:45 (95% CI: 19:00–21:00) compared to those reporting lower eating window times. Differences in pre-bed fasting times were also observed, with a higher proportion of participants with a LEW (89.2%) having 2 h or less of pre-bed fasting (compared to 56.6% in the SEW and 69.5% in the MEW groups). The SEW was associated with a higher proportion of irregular breakfast habits (65.2%), while the MEW and LEW groups had higher proportions of regular breakfast habits (63.0% and 56.5%, respectively). The overall distribution of meals per day in the MEW group was different compared to the SEW group. There were no differences in daily sleep duration, parental BMI, or nationality across eating window status (*p* ≥ 0.097).

The association between daily eating status and BMI-SDS, fat mass index, and waist-to-height ratio is shown in Table 2. The adjusted linear regression analysis revealed that the children in the LEW status had a −0.66 (−1.17 to −0.14) lower BMI-SDS and a −1.06 (−2.04 to −0.09) lower fat mass index score compared to the children in the SEW status. Moreover, the children in the LEW status showed a marginally significant (*p* = 0.057) lower waist-to-height ratio than those in the SEW status (Table 2). No significant difference was observed between children in the MEW and the SEW statuses.

The longitudinal changes in BMI-SDS, fat mass index, and waist-to-height ratio after a healthy intervention are shown in Table 3. No significant interaction between time, school group, and eating window tertiles was observed in BMI-SDS, fat mass index, and waist-to-height ratio (Table 3). A significant difference between school groups was observed for the three outcome variables, and there was a significant effect of eating window tertiles on BMI-SDS (*p* = 0.017) and waist-to-height ratio (*p* = 0.033), which could suggest a positive effect in school group 1 compared to the other school groups, particularly in the group with LEW status, where BMI-SDS changed from −0.61 (−1.34–0.12) to −0.65 (−1.40–0.10), and waist-to-height changed from 0.44 (0.41–0.47) to 0.41 (0.36–0.45). Overall, no evidence was observed for differences in longitudinal BMI-SDS, fat mass index, and waist-to-height ratio changes between school groups and the eating window tertiles combined.

## 4. Discussion

Our study, aiming to analyze meal timing characteristics and their association with obesity-related variables in a non-representative school children sample, found a cross-sectional inverse association between daily eating hours and total and regional body fat markers. Moreover, children with long eating window status tended to have a lower BMI-SDS, fat mass index, and waist-height ratio than their counterparts with short eating window status. However, no impact could be observed in the total and regional body fat markers according to eating window status after a 21-month nutritional education and physical activity intervention.

Overall, this study contributes to describing the characteristics of meal timing variables in school-aged children from Vienna in a real-life context, while previous studies in children have mainly focused on breakfast skipping, the number of daily meals, or late-night eaters [6]. In our study, the median eating window, referred to as a certain period of the day where food intake is limited, was reported to be 11:40 h (95% CI: 9:30–13:30) which aligns with the mean daily eating window reported in a European study (11.5 ± 0.6 h) [12] and a recent meta-analysis covering studies performed in a similar age group [(11.3 h (95% CI 11.0, 11.7)] [7]. The first meal was reported at 7:00 (95% CI: 6:10–9:30) and the last meal at 19:00 (95% CI: 18:00–20:30). These timings slightly differed from those reported for Austrian adults, with median breakfast and last meal being at 7:00 and 18:30, respectively [8], and to those reported previously for an adult study that included Central/Northern European countries [13]. In our study, even in the LEW group, the median last meal (19:45) was reported earlier than the median in a study conducted in Spain (21:07) [14], suggesting that the differences between Western and Central/Northern European countries observed in adults could also be observed in children. These geographical differences explained previously [8,13] could reflect earlier time meals for Austrians compared to those countries in a Western position within their time zone. Added to the geographical aspects, children follow family routines and habits, as they are not yet independent over decisions related to eating.

Meal timing characteristics have been related to internal and environmental characteristics, such as cultural aspects, working activities, and chronotypes, which may vary according to age [4,7]. In children, differences in meal timing characteristics have been reported on weekdays, weekends, and during holidays [4]. Moreover, several studies have shown the heritability of meal timing, particularly for breakfast [4]. Breakfast skipping, one of the meal timing exposures more studied, was reported in our study among the international prevalence rates of 10 to 30% [15]; however, these were lower (16.4%) than rates previously reported for adults (25%) [8]. Regarding breakfast regularity, our study showed differences in eating window status, where a higher proportion of children in the SEW status reported irregular breakfast habits. This difference could be related to the fact that some children have difficulties in establishing morning routines because of lack of time or hunger [15], which can result in eating breakfast irregularly or skipping it, which results in a shorter eating window starting later in the day. A higher caloric intake may also explain a lack of hunger early in the morning [8]; however, we did not assess food intake. Although we found no differences in breakfast regularity between children with overweight/obesity and those with normal weight, a recent systematic review reported that non-daily breakfast consumers (≤6 days/week) had a 45% higher chance of overweight/obesity compared to daily breakfast eaters (7 days/week) [6], which is also supported by other systematic reviews [15,16]. In this sense, the vast differences in the definitions used to assess breakfast regularity could be among the factors explaining the different associations.

The number of meals reported by children in our study was similar (n = 4) to those reported in a European study that included children/adolescents between 8–15 years (n = 4.16) [12]; however, it was higher than those reported for Austrian adults (n = 3) [8], although the authors discussed that the way the exposure was assessed might have determined an underreporting of the number of meals. Nonetheless, a previous report showed three regular meals, with snacking in the afternoon common [17]. In our sample, the pre-bed fasting period reported for more than half of the participants was 1 to 2 h, compared to adults, where the median period was 4.0 (3.0–5.3) hours [8]. In the LEW group, however, more than three-quarters of the participants reported having less than 2 h of fasting before bed, with lower rates than in the SEW group. These longer pre-bed fasting periods in the SEW group may be related to additional eating consumption opportunities and, therefore, higher energy intake, an aspect we did not assess.

A better understanding of the associations of the eating window with health markers may be valuable information to consider within the framework of the current nutritional strategies applied to tackle the childhood obesity problem [18]. Moreover, the acceptability of using a restricted eating window for pediatric weight management has been recently assessed and considered a feasible option accepted by families [19]. Nonetheless, the first step is to describe the current daily eating window according to age and further explore its associations with other meal timing variables and health outcomes. In this sense, this topic has been well-reviewed in adults with excessive body weight, and positive experiences using time-restricted eating windows have shown that weight loss and improved cardiovascular parameters can be achieved [3,4]. However, these strategies still need consideration in children as they have not been widely explored [18]. Our cross-sectional analysis showed that children in the LEW status (≥12:05 h) had lower total and abdominal body fat parameter markers. On the contrary, adults eating their meals for a period of time longer than 12 h had a higher prevalence of abdominal obesity compared to those who ate their meals in a shorter eating duration (≤12 h) [5]. Even among children, the assessment of the role of meal timing with overweight/obesity risk lacks sufficient evidence [20], as the few studies involving children and adolescents are mainly focused on assessing the relationship with an “eating later pattern” [20,21] rather than with daily eating window hours, making comparisons not straightforward. In this regard, a meta-analysis that included mostly cross-sectional studies found no association between eating later and adiposity [21]. However, a recent study in school-aged children from Spain, with median dinner timing at 21:07, found that late-dinner eaters had significantly higher inflammation markers [14]. These differences in how the eating window affects body fat distribution and impacts metabolic health according to age should be further explored. On top of that, it is also unclear how meal timing variables interact with each other and with sleep according to light exposure, and how this could affect health [3,4]. Our results showed shorter sleeping hours in children with overweight/obesity compared to those with normal weight. However, no differences were observed between the timing of the last meal or the regularity of breakfast, two aspects usually related to a shorter sleeping time in children. The latter could have been related to the nature of the data, as we assessed sleeping times and the characteristics of meal timing through self-reports, which are prone to bias. In this sense, shorter sleeping times could be associated with higher calorie intake, poorer diet quality, and less activity because of tiredness [22], aspects that were not assessed in our study. Moreover, the association of sleep timing with adiposity in children, including BMI, has been inconsistent, with some studies reporting no association [23,24] while others showed a negative association [22].

Our longitudinal analysis found no statistically significant impact of the intervention on obesity outcomes across time or interactions with baseline eating window status. While this may be partly due to limited statistical power given the small sample size, it is important to note that the intervention focused on promoting a healthy and varied diet alongside an active lifestyle rather than explicitly targeting or recommending restricted eating windows. Moreover, as mentioned previously, cultural aspects may play a crucial role in children’s food behavior, influencing not only when they eat but also the amount of food consumed at mealtime [7]. The cultural heterogeneity observed in our sample, with high rates of children with migration backgrounds, may explain different eating habits among families (and physical activity patterns), introducing variability in feeding norms or parental perception of adequate nutritional status, which may have influenced the lack of longitudinal changes in our healthy intervention. On the other hand, significant differences were observed between school groups and the tertiles of the eating windows. In the intervention group, children with longer eating windows (≥12:05 h) showed a reduction in BMI-SDS from baseline (−0.61) to follow-up (−0.65), which deserves further exploration. Regarding the waist-to-height ratio, a marker of abdominal adiposity, changes were minimal across all the groups, but the intervention school demonstrated a reduction from 0.44 to 0.41 in children with a LEW status compared to the other groups.

To our knowledge, this is the first study to describe meal timing variables in a non-representative sample of school children from Vienna. The strengths of our study are the objective anthropometric and body composition measurements. However, our study has limitations that should be addressed. First, the data on outcome variables were derived from self-reported questionnaires completed by either parents or caregivers, and information regarding meals did not assess quantity or quality with well-known limitations. The number of meals could not differentiate between main meals and snacks, as participants were asked to report the total daily number of meals consumed. On the other hand, to increase the participation rate, which has proven low in Austria [25], we selected non-invasive, practical tools that allowed us to maintain an acceptable participant burden while collecting multiple data on different concepts. The questions used to assess the meal timing variables were not validated, as, to our knowledge, no validated tool in children was available when our study was conducted. Recently, some studies have validated tools based on image-based [26] and short questionnaires [27], showing they are valid and reliable in accurately reporting meal timing, although in adults.

## 5. Conclusions

Based on a non-representative sample of children from Vienna, our results showed similar timing for breakfast and dinner than Austrian adults, and earlier meal timings than most Western European countries, although similar eating window hours. Overall, our results showed a moderate variation in eating window timing, ranging from 9:30 to 13:30 h. The lower BMI-SDS, fat mass index, and waist-height ratios observed in children with a long eating window status compared to their counterparts with a short eating window status, and the potential effectiveness of the intervention in the subgroup with a long eating window, deserve further exploration of the relationship between meal timing and obesity outcomes in future larger epidemiologic studies, including a representative sample. Moreover, other behavior timing effects, such as sleep and physical activity, which are highly interrelated, should be jointly addressed with the effect of meal timing in children evaluated to provide a broader understanding. Mobile tools and photo diaries are recommended to assess meal timing and diet composition.

## Figures and Tables

**Table 1:** Characteristics of the complete study population (n =138) by tertile of the daily eating window.

	Tertile 1—SEW (≤11:05 h)	Tertile 2—MEW (11:05 to 12:05 h)	Tertile 3—LEW (≥12:05 h)	*p* ^Ɨ^
n	46 (33.3)	46 (33.3)	46 (33.3)	
School 1	17 (37.0)	19 (41.3)	12 (26.1)	0.542
School 2	18 (39.1)	16 (34.8)	18 (39.1)	
School 3	11 (23.9)	11 (23.9)	16 (34.8)	
Sex				
Girls	19 (41.3)	19 (41.3)	17 (37.0)	0.886
Boys	27 (58.7)	27 (58.7)	29 (63.0)	
Age	7.8 (7.2–9.0)	8.2 (7.5–9.1)	7.8 (7.3–9.6)	0.050
Weight status				
Normal weight	31 (67.4)	35 (76.1)	35 (76.1)	0.880
Overweight	7 (15.2)	5 (10.9)	5 (10.9)	
Obesity	8 (17.4)	6 (13.0)	6 (13.0)	
BMI-SDS	0.54 (−1.06–2.58)	0.38 (−0.98–2.83)	−0.04 (−1.29–2.32) ^a^	0.017
BMI	16.8 (14.1–24.9)	16.9 (14.7–26.3)	16.0 (13.8–23.4) ^a^	0.007
Waist-to-height ratio	0.46 (0.41–0.56)	0.44 (0.39–0.57)	0.44 (0.39–0.53)	0.098
Fat mass index	3.99 (2.71–9.18)	3.84 (2.65–9.74)	3.27 (2.27–8.57) ^a^	0.005
FFM/height^2^	13.03 (11.35–15.04)	13.00 (11.41–15.81)	12.60 (11.11–15.15)	0.092
Meal timing variables				
First meal (hh:mm)	8:05 (7:00–10:00)	7:00 (6:30–8:00) ^a^	7:00 (6:00–7:30) ^a^	<0.001
Last meal (hh:mm)	18:00 (17:30–20:00)	19:00 (18:00–20.00) ^a^	19:45 (19:00–21:00) ^a^	<0.001
Eating window (hh:mm/day)	10:30 (8:30–11:00)	11:40 (11:15–12:00) ^a^	12:45 (12:15–13:45) ^a^	<0.001
Pre-bed fasting				
≤1 h	5 (10.9)	6 (13.0)	16 (34.8)	0.001
1–2 h	21 (45.7)	26 (56.5)	25 (54.4)	
2–3 h	14 (30.4)	14 (30.4)	4 (8.7)	
≥3 h	6 (13.0)	0 (0)	1 (2.2)	
Number meals/day	4 (2–5)	4 (3–5) ^a^	4 (3–6)	0.047
Breakfast ^1^				
Regular	16 (34.8)	29 (63.0)	26 (56.5)	0.005
Irregular	30 (65.2)	17 (37.0)	20 (43.5)	
Sleep (hh:mm/day) ^2^	10:00 (8:42–11:00)	9:47 (8:45–10:42)	9:40 (9:00–10:30)	0.240
Parental weight status				
Mother’s BMI, (kg/m^2^) ^3^	24.2 (20.0–36.1)	25.7 (19.8–35.9)	24.7 (20.1–38.7)	0.785
Father’s BMI, (kg/m^2^) ^3^	27.8 (21.8–36.5)	26.9 (21.3–34.7)	27.5 (20.8–34.7)	0.629
Parental background ^4^				
One parent from Austria	9 (20.0)	3 (7.7)	6 (13.6)	0.097
Both parents from Austria	12 (26.7)	12 (30.8)	5 (11.4)	
No parent from Austria	24 (53.3)	24 (61.5)	33 (75.0)	

Values indicate n (%) for categorical variables and median (95% CI) for continuous variables. SEW: short eating window, MEW: moderate eating window, LEW: long eating window. CI: confidence intervals. ^Ɨ^ Chi-square (or Fisher) tests were used for the categorical variables and Kruskal–Wallis test to compare the distributions of the continuous variables across tertiles of the eating window. Models for the continuous variables were adjusted by school group, sex, age, and quantity of sleep. Tests of interaction between tertiles of the eating window and school group were nonsignificant. ^a^ Significant difference compared to tertile 1 of the eating window (*p* < 0.05) in adjusted models, using Tukey’s post hoc test (*p* < 0.05). ^1^ Data available for a total of n = 134 participants. ^2^ Data available for n = 42, n = 35, and n = 42 participants from tertiles 1, 2, and 3, respectively. ^3^ By parental report on weight and height, based on data from n = 125 mothers (n = 44, n = 36, n = 45 for tertiles 1, 2, 3, respectively) and n = 114 fathers (n = 39, n = 33, n = 42 for tertiles 1, 2, 3, respectively). ^4^ As assessed by parents’ nationality, based on data from n = 128.

**Table 2:** Association between tertiles of the daily eating window, BMI-SDS, fat mass index, and waist-to-height ratio in the complete study population (n = 138).

Outcome (SEW as Ref.)	Crude β (95% CI)	*p*	Adjusted β (95% CI)	*p*
BMI-SDS				
MEW	−0.20 (−0.69–0.29)	0.429	−0.06 (−0.60–0.47)	0.816
LEW	−0.56 (−1.05 to −0.07)	0.026	−0.66 (−1.17 to −0.14)	0.013
Fat mass index				
MEW	−0.19 (−1.14–0.75)	0.687	0.03 (−0.99–1.04)	0.959
LEW	−0.79 (−1.74–0.16)	0.104	−1.06 (−2.04 to −0.09)	0.032
Waist-to-height ratio				
MEW	−0.01 (−0.03–0.01)	0.332	−0.01 (−0.03–0.02)	0.582
LEW	−0.02 (−0.04–0.00)	0.092	−0.02 (−0.04–0.00)	0.057

Ref: reference. SEW: short eating window (tertile 1); MEW: moderate eating window (tertile 2); LEW: long eating window (tertile 3). Values indicate beta coefficients (β) and 95% confidence interval. Multivariable linear regression analysis was performed considering SEW as the reference category. Adjusted model included adjustments for school group, age, sex, and quantity of sleep.

**Table 3:** Changes in BMI-SDS, fat mass index, and waist-to-height ratio after a healthy intervention.

	Tertile 1—SEW		Tertile 2—MEW		Tertile 3—LEW		F-Value, (*p*-Value)			
	Baseline	Last Follow-Up	Baseline	Last Follow-Up	Baseline	Last Follow-Up	Time	School Group	Tertiles of EW	Interaction ^Ɨ^
BMI-SDS							0.05 (0.819)	3.21 (0.044)	4.24 (0.017)	0.63 (0.813)
School 1	0.71 (0.07–1.35)	0.63 (0.003–1.26)	0.35 (−0.24–0.94)	0.36 (−0.28–1.01)	−0.61 (−1.34–0.12)	−0.65 (−1.40–0.10)				
School 2	0.64 (0.001–1.28)	0.53 (−0.08–1.14)	0.46 (−0.25–1.17)	0.27 (−0.45–0.99)	0.40 (−0.27–1.07)	0.38 (−0.22–0.97)				
School 3	0.87 (0.07–1.68)	0.74 (−0.01–1.49)	1.29 (0.44–2.15)	1.30 (0.36–2.25)	0.38 (0.44–2.15)	0.33 (−0.40–1.07)				
Fat mass index							1.07 (0.304)	5.51 (0.005)	2.60 (0.078)	1.20 (0.291)
School 1	5.01 (3.81–6.22)	4.52 (3.16–5.88)	4.77 (3.67–5.88)	4.52 (3.15–5.88)	3.08 (1.71–4.44)	2.39 (0.77–4.01)				
School 2	4.97 (3.77–6.17)	4.16 (2.84–5.48)	4.63 (3.30–5.97)	3.62 (2.07–5.18)	4.68 (3.43–5.92)	4.19 (2.90–5.49)				
School 3	6.25 (4.75–7.76)	5.11 (3.47–6.74)	6.71 (5.11–8.31)	6.86 (4.84–8.89)	5.18 (3.99–6.37)	4.88 (3.34–6.43)				
Waist-to-height ratio							0.13 (0.715)	4.26 (0.016)	3.50 (0.033)	1.33 (0.209)
School 1	0.48 (0.45–0.51)	0.47 (0.44–0.51)	0.46 (0.43–0.48)	0.47 (0.44–0.51)	0.44 (0.41–0.47)	0.41 (0.36–0.45)				
School 2	0.46 (0.44–0.49)	0.48 (0.45–0.52)	0.46 (0.43–0.49)	0.46 (0.42–0.50)	0.45 (0.42–0.48)	0.46 (0.43–0.50)				
School 3	0.48 (0.45–0.52)	0.51 (0.47–0.56)	0.50 (0.47–0.54)	0.51 (0.36–0.57)	0.47 (0.45–0.50)	0.47 (0.43–0.52)				

EW: eating window. Values indicate least squares means (95% CI) at baseline and after 21 months (follow-up) according to school group and tertiles of the EW (SEW: short eating window; MEW: moderate eating window; LEW: long eating window). Mixed models for repeated measures were employed. The factors school group (school 1, school 2, school 3), time (baseline, last follow-up), and tertiles of the EW (tertile 1, tertile 2, tertile 3), and their interaction, were treated as fixed effects. Participants were treated as random effects to account for potential clustering. Covariates considered included age, sex, and quantity of sleep. ^Ɨ^ Interaction for group × time × tertile EW. Tests for two-level interaction were nonsignificant. School 1: tertile 1 n = 17; tertile 2 n = 19; tertile 3 n = 12. School 2: tertile 1 n = 18; tertile 2 n = 15; tertile 3 n = 18. School 3: tertile 1 n = 11; tertile 2 n = 11; tertile 3 n = 1.

## Data Availability

The data presented in this study are available on request from the corresponding author due to ethical reasons.

## Supplementary Materials

The following supporting information can be downloaded at: https://www.mdpi.com/article/10.3390/nu17101661/s1, Table S1: General characteristics of the complete population study (n = 138) by sex; Table S2: General characteristics of the participants by school group; Table S3: Meal timing variables according to weight status in the complete study population (n = 138).

## References

[1] Lobstein T.A., Powis J., Jackson-Leach R. (2024). World Obesity Atlas 2024. https://www.worldobesity.org/.

[2] Rubino F., Cummings D.E., Eckel R.H., Cohen R.V., Wilding J.P.H., Brown W.A., Stanford F.C., Batterham R.L., Farooqi I.S., Farpour-Lambert N.J. (2025). Definition and diagnostic criteria of clinical obesity. Lancet Diabetes Endocrinol..

[3] Chaix A., Manoogian E.N., Melkani G.C., Panda S. (2019). Time-Restricted Eating to Prevent and Manage Chronic Metabolic Diseases. Annu. Rev. Nutr..

[4] Peters B., Vahlhaus J., Pivovarova-Ramich O. (2024). Meal timing and its role in obesity and associated diseases. Front. Endocrinol..

[5] da Cunha N.B., Teixeira G.P., Rinaldi A.E.M., Azeredo C.M., Crispim C.A. (2023). Late meal intake is associated with abdominal obesity and metabolic disorders related to metabolic syndrome: A chrononutrition approach using data from NHANES 2015–2018. Clin. Nutr..

[6] Fiore G., Scapaticci S., Neri C.R., Azaryah H., Escudero-Marín M., Pascuzzi M.C., La Mendola A., Mameli C., Chiarelli F., Campoy C. (2023). Chrononutrition and metabolic health in children and adolescents: A systematic review and meta-analysis. Nutr. Rev..

[7] Townley J., Northstone K., Hinton E.C., Hamilton-Shield J., Searle A., Leary S. (2024). Daily Duration of Eating for Children and Adolescents: A Systematic Review and Meta-Analysis. Nutrients.

[8] Santonja I., Bogl L.H., Degenfellner J., Klösch G., Seidel S., Schernhammer E., Papantoniou K. (2023). Meal-timing patterns and chronic disease prevalence in two representative Austrian studies. Eur. J. Nutr..

[9] Stadt Wien (2023). Wirtschaft A und S. Bezirke in Zahlen—Statistiken. statistik.wien.gv.at. Wien. https://www.wien.gv.at/statistik/bezirke/index.html.

[10] Kromeyer-Hauschild K., Wabitsch M., Kunze D., Geller F., Geiß H.C., Hesse V., Von Hippel A., Jaeger U., Johnsen D., Korte W. (2001). Perzentile fr den Body-Mass-Index fr das Kindes-und Jugendalter Unter Heranziehung Verschiedener Deutscher Stichproben. Monatsschr. Kinderheilkd.

[11] Reynolds A.M., Spaeth A.M., Hale L., Williamson A.A., LeBourgeois M.K., Wong S.D., Hartstein L.E., Levenson J.C., Kwon M., Hart C.N. (2023). Pediatric sleep: Current knowledge, gaps, and opportunities for the future. Sleep.

[12] Intemann T., Bogl L.H., Hunsberger M., Lauria F., De Henauw S., Molnár D., Moreno L.A., Tornaritis M., Veidebaum T., Ahrens W. (2024). A Late Meal Timing Pattern Is Associated with Insulin Resistance in European Children and Adolescents. Pediatr. Diabetes.

[13] Huseinovic E., Winkvist A., Freisling H., Slimani N., Boeing H., Buckland G., Schwingshackl L., Olsen A., Tjønneland A., Stepien M. (2018). Timing of eating across ten European countries—Results from the European Prospective Investigation into Cancer and Nutrition (EPIC) calibration study. Public Health Nutr..

[14] Martínez-Lozano N., Tvarijonaviciute A., Ríos R., Barón I., Scheer F.A.J.L., Garaulet M. (2020). Late Eating Is Associated with Obesity, Inflammatory Markers and Circadian-Related Disturbances in School-Aged Children. Nutrients.

[15] Monzani A., Ricotti R., Caputo M., Solito A., Archero F., Bellone S., Prodam F. (2019). A Systematic Review of the Association of Skipping Breakfast with Weight and Cardiometabolic Risk Factors in Children and Adolescents. What Should We Better Investigate in the Future?. Nutrients.

[16] Ardeshirlarijani E., Namazi N., Jabbari M., Zeinali M., Gerami H., Jalili R.B., Larijani B., Azadbakht L. (2019). The link between breakfast skipping and overweigh/obesity in children and adolescents: A meta-analysis of observational studies. J. Diabetes Metab. Disord..

[17] Kiefer I., Haberzettl C., Rieder C. (2000). Ernährungsverhalten und Einstellung zum Essen der ÖsterreicherInnen. J. Ernährungsmedizin.

[18] Bakhsh J., Salvy S.-J., Vidmar A.P. (2024). Intermittent fasting as a treatment for obesity in young people: A scoping review. npj Metab. Health Dis..

[19] Tucker J.M., Siegel R., Murray P.J., Han J.C., Boyer K., Reed N., Allenby T., Novick M. (2022). Acceptability of Time-Limited Eating in Pediatric Weight Management. Front. Endocrinol..

[20] Saltaouras G., Kyrkili A., Bathrellou E., Georgoulis M., Yannakoulia M., Bountziouka V., Smrke U., Dimitrakopoulos G., Kontogianni M.D. (2024). Associations between Meal Patterns and Risk of Overweight/Obesity in Children and Adolescents in Western Countries: A Systematic Review of Longitudinal Studies and Randomised Controlled Trials. Children.

[21] Zou M., Northstone K., Perry R., Johnson L., Leary S. (2022). The association between later eating rhythm and adiposity in children and adolescents: A systematic review and meta-analysis. Nutr. Rev..

[22] Felső R., Lohner S., Hollódy K., Erhardt É., Molnár D. (2017). Relationship between sleep duration and childhood obesity: Systematic review including the potential underlying mechanisms. Nutr. Metab. Cardiovasc. Dis..

[23] Thumann B.F., Buck C., De Henauw S., Hadjigeorgiou C., Hebestreit A., Lauria F., Lissner L., Molnár D., Moreno L.A., Veidebaum T. (2021). Cross-sectional associations between objectively measured sleep characteristics and body mass index in European children and adolescents. Sleep Med..

[24] Dutil C., Podinic I., Sadler C.M., da Costa B.G., Janssen I., Ross-White A., Saunders T.J., Tomasone J.R., Chaput J.-P. (2022). Sleep timing and health indicators in children and adolescents: A systematic review. Health Promot. Chronic Dis. Prev. Can..

[25] WHO Regional Office for Europe (2023). Childhood Obesity Surveillance Initiative (COSI)—Bericht Österreich 2020.

[26] Ramírez-Contreras C., Farran-Codina A., Zerón-Rugerio M.F., Izquierdo-Pulido M. (2023). Relative Validity and Reliability of the Remind App as an Image-Based Method to Assess Dietary Intake and Meal Timing in Young Adults. Nutrients.

[27] Chakradeo P., Rasmussen H.E., Swanson G.R., Swanson B., Fogg L.F., Bishehsari F., Burgess H.J., Keshavarzian A. (2021). Psychometric Testing of a Food Timing Questionnaire and Food Timing Screener. Curr. Dev. Nutr..

