# Supplementary material for: Daily Eating Window and Obesity Markers in a Sample of Schoolchildren from Vienna: Insights from the EDDY Study
Source: Nutrients. 2025 May 13;17(10):1661. doi: 10.3390/nu17101661 (PMC12114318; doi:10.3390/nu17101661)
Supplement: Supplementary file 1 [file nutrients-17-01661-s001.zip › nutrients-3598030-supplementary.pdf]

Supplementary Material

Daily Eating Window and Obesity Markers in a Sample of Schoolchildren from Vienna: Insights from the EDDY Study

Table S1. General characteristics of the complete population study (n = 138) by sex

	**Girls**	**Boys**	***p*** [ⱡ]
n	55 (39.9)	83 (60.1)	
Age (years)	7.9 (7.2-9.0)	8.0 (7.3-9.3)	0.218
Anthropometric variables			
BMI (kg/m^2)	16.8 (13.8-26.3)	16.3 (14.1-23.0)	0.458
BMI-SDS	0.41 (−1.00-2.83)	0.21 (−1.25-2.38)	0.317
Weight status			
Normal weight	40 (72.7)	61 (73.5)	0.032
Overweight	3 (5.5)	14 (16.9)	
Obesity	12 (21.8)	8 (9.6)	
Waist-to-height ratio	0.45 (0.39-0.57)	0.45 (0.40-0.54)	0.867
Fat mass index	4.00 (2.77-9.81)	3.37 (2.45-8.66)	0.003
Meal timing variables			
First meal (hh:mm)	7:15 (6:00-9:30)	7:00 (6:20-9:30)	0.414
Last meal (hh:mm)	19:00 (18:00-20:45)	19:00 (18:00-20:00)	0.779
Eating window (hh:mm /day)	11:39 (9:00-13:45)	11:40 (9:00-13:15)	0.767
Pre-bed fasting			
≤ 1 hour	8 (14.6)	19 (22.9)	0.454
1-2 hours	28 (50.9)	44 (53.0)	
2-3 hours	16 (29.1)	16 (19.3)	
≥ 3 hours	3 (5.4)	4 (4.8)	
Number meals/day	4 (3-5)	4 (3-6)	0.947
Breakfast [1]			
Regular	26 (47.3)	45 (54.2)	0.424
Irregular	29 (52.7)	38 (45.8)	
Sleep (hh:mm/day) [2]	10:00 (8:55-10:47)	9:38 (8:42-11:00)	0.264
Parental weight status			
Mother's BMI, (kg/m^2) [3]	25.4 (19.8-36.8)	24.2 (20.0-36.5)	0.047
Father's BMI, (kg/m^2) [3]	28.3 (21.8-36.3)	27.2 (20.8-35.0)	0.496
Parental background [4]			
One parent from Austria	7 (13.5)	11 (14.5)	0.445
Both parents from Austria	9 (17.3)	20 (26.3)	
No parent from Austria	36 (69.2)	45 (59.2)	

Values indicate n (%) for categorical variables and median (95 % CI) for continous variables. CI: confidence intervals. [ⱡ] Chi-square (or Fisher) tests were used for categorical variables and Mann-Whitney U test to compare the distributions of continous variables across sex categories. [1] Data available for a total of n = 134 participants. [2] Data available for a total of n =119 participants. [3] By parental report on weight and height, based on data from n = 125 mothers and n = 114 fathers. [4] As assessed by parents' nationality, based on data from n = 128.

Table S2. General characteristics of the participants by school group

	**School 1**	**School 2**	**School 3**	***p*** [ǂ]
n	48	52	38	
Age (years)	8.1 (7.2-9.1)	7.8 (7.2-9.0)	8.1 (7.3-9.6)	0.090
Sex				0.678
Girls	17 (35.4)	21 (40.4)	17 (44.7)	
Boys	31 (64.6)	31 (59.6)	21 (55.3)	
BMI (kg/m^2)	16.1 (13.6-24.9)	16.5 (14.0-23.0)	17.2 (14.1-27.1)	0.138
SDS-BMI	0.01 (−1.57-2.83)	0.37 (−1.00-2.38)	0.58 (−1.09-2.71)	0.162
Weight status				0.050
Normal weight	39 (81.3)	39 (75.0)	23 (60.5)	
Overweight	5 (10.4)	8 (15.4)	4 (10.5)	
Obesity	4 (8.3)	5 (9.6)	11 (29.0)	
Waist-to-height ratio	0.45 (0.39-0.56)	0.44 (0.39-0.54)	0.46 (0.41-0.56)	0.017
Fat mass index	3.37 (2.19-9.00)	3.66 (2.64-8.66)	4.14 (2.63-10.80)	0.009
Meal timing variables				
First meal (hh:mm)	7:00 (6:10-9:30)	7:00 (6:40-9:30)	7:15 (6:00-9:00)	0.995
Last meal (hh:mm)	19:00 (18:00-20:30)	19:00 (18:00-20:20)	19:00 (18:00-21:00)	0.501
Eating window (hh:mm /day)	11:35 (9:30-13:00)	11:42 (8:30-13:15)	12:00 (9:00-13:45)	0.330
Pre-bed fasting				0.403
≤ 1 hour	9 (18.8)	14 (26.9)	4 (10.5)	
1-2 hours	27 (56.3)	25 (48.1)	20 (52.6)	
2-3 hours	9 (18.8)	12 (23.1)	11 (29.0)	
≥ 3 hours	3 (6.3)	1 (1.9)	3 (7.9)	
Number meals/day	4 (3-6)	4 (3-6)	4 (3-5)	0.011
Breakfast [1]				0.073
Regular	31 (64.6)	24 (46.2)	16 (42.1)	
Irregular	17 (35.4)	28 (53.9)	22 (57.9)	
Sleep (hh:mm/day) [2]	9:38 (8:42-10:42)	9:49 (8:42-10:47)	9:53 (8:34-11:00)	0.359
Parental weight status				
Mother's BMI, (kg/m^2) [3]	25.1 (20.0-36.1)	24.5 (20.1-35.4)	24.4 (18.8-38.7)	0.793
Father's BMI, (kg/m^2) [3]	26.2 (21.3-32.9)	28.4 (23.0-34.7)	29.1 (20.6-38.6)	0.033
Parental background [4]				0.096
One parent from Austria	5 (11.1)	9 (18.4)	4 (11.8)	
Both parents from Austria	9 (20.0)	16 (32.6)	4 (11.8)	
No parent from Austria	31 (68.9)	24 (49.0)	26 (76.5)	

Values indicate n (%) for categorical variables or median (95 % CI) for continous variables. CI: confidence intervals. [ǂ] Chi-square (or Fisher) tests were used for categorical variables and Kruskal-Wallis test to compare the distributions of continous variables across school groups. [1] Data available for a total of n = 134 participants. [2] Data available for a total of n = 119 participants (n = 45, n = 46 and n = 28 participants from school 1, school 2 and school 3, respectively). [3] By parental report on weight and height, based on data from n = 125 and n = 114 fathers. [4] As assessed by parents' nationality, based on data from n = 128.

Table S3. Meal timing variables according to weight status in the complete study population (n =138)

	**Normal weight**	**Overweight/Obesity**	*p* [ɫ]
	101 (73.2)	37 (26.8)	
Age	7.9 (7.3-9.3)	8.0 (7.2-9.6)	0.645
Meal timing variables			
First meal (hh:mm)	7:15 (6:10-9:00)	7:00 (6:30-10:00)	0.377
Last meal (hh:mm)	19:00 (18:00-20:00)	19:00 (18:00-20:45)	0.158
Eating window (hh:mm/day)	11:40 (9:30-13:20)	11:41 (8:00-13:45)	0.440
Pre-bed fasting			
≤ 1 hour	21 (20.8)	6 (16.2)	0.081
1-2 hours	57 (56.4)	15 (40.5)	
2-3 hours	20 (19.8)	12 (32.4)	
≥ 3 hours	3 (3.0)	4 (10.8)	
Number meals/day	4 (3-6)	4 (2-5)	0.031
Breakfast [1]			
Regular	56 (55.5)	15 (40.5)	0.121
Irregular	45 (44.5)	22 (59.5)	
Sleep (hh:mm/day) [2]	10:00 (8:55-10:55)	9:30 (8:28-11:00)	0.005

Values indicate n (%) for categorical variables and median (95 % CI) for continous variables. CI: confidence intervals. [ɫ] Chi-square (or Fisher) tests were used for categorical variables and Mann-Whitney U test to compare the distributions of continous variables across weight status categories. Models for continous variables were adjusted by school group, sex, age and quantity of sleep. Test of interaction between weight status categories and school group was nonsignificant. [1] Data available for a total of n = 134 participants. [2] Data available for a total of n = 119 participants.
